# Comparative Study of the Cytotoxic Effects of Outer Membrane Vesicles from *Proteus mirabilis* and *Escherichia coli* on HK-2 Cells

**DOI:** 10.3390/pathogens15080859

**Published:** 2026-08-18

**Authors:** Qingchen Du, Xijie Ding, Endi Zhang, Xinyang Niu, Guojun Chen, Chaoyue Ji, Weiguo Hu

**Affiliations:** 1 Department of Urology, Beijing Tsinghua Changgung Hospital, School of Clinical Medicine, Tsinghua Medicine, Tsinghua University, Beijing 102218, China; duqingchen2026@163.com (Q.D.); dxj320831@163.com (X.D.); zhangendi328@163.com (E.Z.); nxy25@mails.tsinghua.edu.cn (X.N.); jcya02468@btch.edu.cn (C.J.); 2 Department of Urology, Qinghai University Affiliated Hospital, School of Clinical Medicine, Qinghai University, Xining 810000, China; chenguojun68@126.com

**Keywords:** outer membrane vesicles, urinary tract infection, uropathogenic *Escherichia coli*, *Proteus mirabilis*, oxidative stress, cell injury

## Abstract

**Objectives:** This study aimed to explore the cellular injury phenotypes induced by outer membrane vesicles (OMVs) from uropathogenic *Escherichia coli* (*E. coli*, UPEC) and *Proteus mirabilis* (*P. mirabilis*, *P. m*) in human renal tubular epithelial HK-2 cells in vitro. **Introduction:** In urological practice, UPEC and *P. mirabilis* are representative pathogens of uncomplicated and complicated UTIs, respectively. Both are Gram-negative bacteria, and a notable feature of these bacteria is their ability to secrete OMVs. OMVs are nanoscale vesicles containing lipopolysaccharides (LPS), phospholipids, peptidoglycan, nucleic acids, and various virulence factors such as proteases and toxins. OMVs serve as effective carriers, delivering these toxic substances to host cells, triggering inflammatory responses, cellular damage, and ultimately cell death. **Methods:** In this study, we cultured standard pathogenic strains of *E. coli* and *P. mirabilis* in lysogeny broth (LB) medium until they reached the logarithmic growth phase. OMVs were isolated and identified. The uptake of OMVs by HK-2 cells was observed in vitro, and we evaluated the cytotoxic effects of both types of OMVs by measuring cell viability, oxidative stress, membrane permeability, mitochondrial function, and organelle morphology. **Results:** The results demonstrated that OMVs from both bacterial strains were efficiently internalized by HK-2 cells. Both OMVs induced oxidative stress, decreased antioxidant capacity, disrupted membrane permeability, and damaged mitochondrial function, leading to cell injury. Interestingly, while the two types of OMVs caused comparable cytotoxicity in terms of cell viability, oxidative stress and mitochondrial membrane potential, they produced distinct patterns of mitochondrial ultrastructural injury: *P. mirabilis* OMVs primarily caused mitochondrial structural deformation and blurred cristae, while *E. coli* OMVs led to mitochondrial swelling, cristae breakage, and vacuolization. These findings provide phenotypic evidence and experimental clues for understanding OMV-associated renal tubular epithelial injury caused by different uropathogens.

## 1. Introduction

Urinary tract infections (UTIs) are among the most common infectious diseases in humans, affecting approximately 400 million people worldwide. The incidence of UTIs has been rising annually, making it a significant global public health concern [1]. Uropathogenic *Escherichia coli* (UPEC) is the most prevalent pathogen causing UTIs, responsible for 80–90% of community-acquired and 30–50% of hospital-acquired urinary tract infections [2]. Additionally, *Proteus mirabilis*, due to its ability to form crystalline biofilms, has gained attention for its role in complicated catheter-associated urinary tract infections [3]. Both UPEC and *P. mirabilis* are Gram-negative bacteria.

One important biological characteristic of Gram-negative bacteria is their ability to secrete OMVs. OMVs are nanoscale, bilayered spherical vesicles formed by the shedding of the bacterial outer membrane, with diameters ranging from 85 to 260 nm [4]. In recent years, OMVs have gained increasing attention as a significant mediator of interactions between bacteria and host cells [5,6]. OMVs act as biological carriers, encapsulating and transporting various bioactive components, including proteins, lipids, and nucleic acids, thereby mediating complex interactions between bacteria and host cells. They play a crucial role in bacterial infection, immune responses, and inflammatory damage [7,8]. Therefore, OMVs are considered vital tools for bacterial invasion and interference with host cell functions [9]. Existing studies have shown that OMVs are internalized by host cells, delivering their cargo and inducing pathological changes, such as oxidative stress, mitochondrial dysfunction, and increased cell membrane permeability, ultimately leading to cell injury or apoptosis [10,11].

Both *Proteus mirabilis* and *Escherichia coli* are classic Gram-negative bacteria, and their OMVs play a critical role in inducing host cell damage [12]. Notably, renal tubular epithelial cell injury induced by uropathogens is also considered a key initiating factor for urinary tract stone formation [13]. Although there are significant similarities in the physicochemical properties and cytoplasmic proteins of OMVs from *P. mirabilis* and *E. coli*, there are differences in the abundance of virulence components [14]. Renal tubular epithelial cell injury is a central factor in the formation and development of urinary tract stones [15,16]. Studies have shown that after renal tubular epithelial cell injury, changes occur in the cell surface microstructure, and tight junctions between cells are disrupted. This exposes the damaged basement membrane to urine, creating a favorable environment for the aggregation and growth of stone crystals [17,18].

At present, research on bacterial OMVs is advancing, but there is still a lack of systematic comparative analysis of the biological effects of OMVs from different urinary pathogens on the same host cell. In this study, using human kidney tubular epithelial (HK-2) cells as an in vitro model, we isolated and identified OMVs from *P. mirabilis* and *E. coli* and compared their cytotoxic effects on HK-2 cells by evaluating cell viability, oxidative stress levels, membrane permeability, mitochondrial function, and ultrastructural changes in organelles after 12 h treatment. This phenotype-focused comparative study was designed to identify the shared and distinct effects of the two OMV preparations, provide experimental clues for subsequent compositional analyses and mechanistic validation, and lay a theoretical foundation for the development of targeted intervention strategies to mitigate urinary tract infection-associated renal damage.

## 2. Materials and Methods

### 2.1. Bacterial Strains and Culture

Standard strains of *Proteus mirabilis* (strain number B80977 = ATCC 43071) and uropathogenic *Escherichia coli* (UPEC, *E. coli* O6:H1, strain number B84186 = ATCC 700928) were purchased. The bacterial strains were inoculated onto LB agar plates and incubated in a constant-temperature incubator (37 °C). After 24 h, colonies were picked and incubated in lysogeny broth (LB) medium at a constant temperature of 37 °C until the bacteria reached the logarithmic growth phase, after which they were reserved for subsequent use.

### 2.2. Isolation and Characterization of Outer Membrane Vesicles (OMVs)

OMVs were isolated following the method established by Kim et al. (2017) [19]. LB medium cultures of the bacteria were harvested and centrifuged at 7000× *g* for 60 min to obtain the supernatant of each bacterial strain. The supernatant was filtered sequentially through 0.45 µm and 0.22 µm Millipore filters to remove bacterial cells and debris. The filtered supernatant was transferred to ultracentrifuge tubes (BeckmanCoulter, Brea, CA, USA), balanced, and subjected to ultracentrifugation at 160,000× *g* for 3 h at 4 °C using a Himac ultracentrifuge (Eppendorf Himac Technologies Co., Ltd., Hitachinaka, Ibaraki, Japan). After centrifugation, the supernatant was discarded, and the pellet was gently resuspended in phosphate-buffered saline (PBS). The OMV preparations were subsequently purified by size-exclusion chromatography (SEC), and the purified *P. mirabilis*-OMVs and *E. coli*-OMVs were stored at −80 °C. The absence of viable bacteria was confirmed by plating on LB agar and incubation at 37 °C for 48 h. The protein concentrations of the OMVs were determined using a BCA protein assay kit (EpiZyme, Shanghai, China, ZJ101). The morphology of the vesicles was observed by Transmission Electron Microscopy (TEM), and nanoparticle tracking analysis (NTA) was used to measure the particle size distribution. OMV yield was quantified by protein mass (µg/mL). To ensure biological comparability between groups, particle number normalization (particles/mL) was performed after concentration determination. Since the particle size and protein content of the two OMVs were similar, protein concentration was used for unified dosing, and particle number was verified for consistency.

### 2.3. Cell Culture and Reagents

Human kidney proximal tubular HK-2 cells (Procell, Wuhan, China) were cultured in Minimum Essential Medium (MEM, Gibco; Thermo Fisher Scientific, Waltham, MA, USA) supplemented with 10% fetal bovine serum (FBS, Invitrogen; Thermo Fisher Scientific), 5 mM sodium pyruvate (Gibco; Thermo Fisher Scientific), and 1% penicillin-streptomycin (Gibco; Thermo Fisher Scientific) at 37 °C with 5% CO_2_ in a humidified incubator. HK-2 cells within 20 passages were used for all experiments to avoid phenotypic changes caused by long-term subculture.

### 2.4. Endocytosis of OMVs by HK-2 Cells

To determine whether OMVs from both bacteria could be effectively internalized by HK-2 cells, a cell uptake experiment was conducted using PKH67 green fluorescence dye. OMVs were extracted as described above. The following reagents were used: PKH67 green fluorescent dye (MINI67, Sigma-Aldrich, St. Louis, MO, USA), DAPI nuclear stain, phalloidin red fluorescent stain for microfilaments (C2235-20 mL, Beyotime Biotechnology, Shanghai, China), PBS, 0.25% trypsin, 4% paraformaldehyde, and 1% BSA-PBS blocking solution. OMVs were labeled by resuspending 10 µg OMVs in 100 µL serum-free medium and adding 8 µL of PKH67 staining solution. The mixture was incubated at room temperature for 30 min in the dark. After incubation, 1% BSA-PBS was added to quench the unbound dye, and the cells were co-cultured with the labeled OMVs. The HK-2 cells were seeded at a density of 1 × 10^5^ cells per dish in confocal culture plates and incubated for 24 h to allow attachment. Labeled OMVs were added, and the cells were further incubated. DAPI solution (1 µg/mL) was then added to stain the nuclei for 5 min at room temperature in the dark. The cells were gently washed twice with pre-chilled PBS and analyzed using a laser scanning confocal microscope to capture fluorescence images. DAPI-stained nuclei, PKH67-labeled OMVs, and phalloidin-stained microfilaments were visualized in the blue, green, and red fluorescence channels, respectively. Images from the three channels were merged to evaluate the intracellular distribution of OMVs in HK-2 cells. Fluorescence quantitative analysis was conducted with ImageJ software1.54 (National Institutes of Health, Bethesda, MD, USA). to quantify the normalized intracellular PKH67 fluorescence intensity of OMVs at various incubation durations. Briefly, the total PKH67 fluorescence intensity was normalized to the nuclear DAPI fluorescence intensity to exclude analytical biases arising from inconsistent cell density among visual fields.

### 2.5. Cell Viability Assay

Cell viability was assessed using the CCK-8 (Enhanced Cell Counting Kit-8, Bioss, Beijing, China, BA00208) assay. HK-2 cells were seeded in 96-well plates and treated with different concentrations (5, 10, 20, 30, and 45 µg/mL) of *P. mirabilis*-OMVs and *E. coli*-OMVs for 3, 6, and 12 h to evaluate concentration- and time-dependent effects. After treatment, the CCK-8 reagent was added according to the manufacturer’s instructions, and the cells were incubated at 37 °C. Absorbance at 450 nm was measured using a BioTek/H1MF multifunctional microplate reader to calculate cell viability. The treatment concentration and duration used in the subsequent multi-parameter assays were selected on the basis of these screening results.

### 2.6. Detection of Intracellular Reactive Oxygen Species (ROS) Levels

Intracellular ROS levels were measured using the DCFH-DA fluorescence probe method. HK-2 cells were seeded in culture plates and incubated for 8 h. After the cells adhered, 45 µg/mL *P. mirabilis*-OMVs and *E. coli*-OMVs were added, and co-incubation was performed for 12 h. After removing the culture medium, cells were incubated with 10 µM DCFH-DA (Beyotime Biotechnology, Shanghai, China) at 37 °C for 30 min in the dark. The cells were washed twice with pre-chilled PBS to remove any unbound probe. ROS levels were assessed by measuring fluorescence intensity using the EVOS M7000 live cell imaging system (Thermo Fisher Scientific, Waltham, MA, USA) at excitation and emission wavelengths of 488 nm and 525 nm, respectively.

### 2.7. Oxidative Stress-Related Marker Assays

After a 12 h treatment with OMVs, HK-2 cells were collected, and oxidative stress markers were measured using commercially available assay kits. Malondialdehyde (MDA) levels were determined using an MDA assay kit (A003-1-2, Jiancheng, Nanjing, China). Total superoxide dismutase (SOD) activity was measured using a total SOD activity assay kit (S0101M, Beyotime Biotechnology, Shanghai, China). Catalase (CAT) activity was determined using a catalase activity assay kit (S0051, Beyotime Biotechnology, Shanghai, China).

### 2.8. Measurement of Cell Membrane Permeability

Cell membrane permeability was assessed by measuring lactate dehydrogenase (LDH) release using an LDH assay kit (A020-2-2, Jiancheng, Nanjing, China). After treatment with *P. mirabilis*-OMVs and *E. coli*-OMVs for 12 h, cell culture supernatants were collected. LDH activity was measured according to the manufacturer’s instructions, and the absorbance was determined at 450 nm using a microplate reader.

### 2.9. Mitochondrial Membrane Potential (MMP) Detection

Mitochondrial membrane potential was assessed using the JC-1 enhanced mitochondrial membrane potential detection kit (C2003S, Beyotime Biotechnology, Shanghai, China). After 12 h of OMV treatment, the culture medium was removed, and 1 mL of JC-1 staining solution was added to the cells. The cells were incubated at 37 °C for 30 min in the dark. After staining, the cells were washed twice with JC-1 staining buffer and analyzed using the EVOS M7000 live cell imaging system. In healthy cells with intact mitochondrial membrane potential, JC-1 aggregates emit red fluorescence; in damaged cells with reduced mitochondrial membrane potential, JC-1 exists as monomers and emits green fluorescence. The red-to-green fluorescence ratio was used to evaluate mitochondrial membrane potential changes.

### 2.10. Transmission Electron Microscopy (TEM)

HK-2 cells were seeded in 35 mm culture dishes and treated with 45 µg/mL *P. mirabilis*-OMVs or *E. coli*-OMVs for 12 h. The cells were fixed with 2.5% glutaraldehyde at 4 °C for 2 h. The samples were dehydrated through a gradient series of ethanol, followed by overnight infiltration with EMbed 812 embedding medium. After embedding, the samples were baked in a 60 °C oven for 48 h. Thin sections (60–80 nm) were prepared using an ultramicrotome, stained with uranyl acetate, and examined under a Hitachi HT7800 transmission electron microscope (Tokyo, Japan) at an accelerating voltage of 80 kV. The ultrastructural changes in mitochondria and other subcellular organelles were recorded. At least 10 mitochondria per group were randomly selected for quantitative analysis using ImageJ. Mitochondrial boundaries were manually traced to calculate mitochondrial area, and cristae number density was defined as the number of cristae divided by mitochondrial area. Statistical analyses were performed using GraphPad Prism 10.0. Data normality was assessed using the Shapiro–Wilk test. For normally distributed data, multiple-group comparisons were performed using one-way ANOVA followed by Tukey’s post hoc test for pairwise comparisons.

### 2.11. Statistical Analysis

Data analysis was carried out with GraphPad Prism 10 (GraphPad, San Diego, CA, USA). Data are shown as mean ± SD. Before statistical analysis, the normality of data distribution and homogeneity of variance were verified. One-way or two-way ANOVA followed by Tukey’s post hoc multiple comparison test was used for comparisons among multiple groups, and Student’s *t*-test was applied for two-group comparisons. All experiments were repeated at least three times (*n* = 3) unless otherwise noted. A *p* value < 0.05 indicated statistical significance. Significance markers: * *p* < 0.05, ** *p* < 0.01, *** *p* < 0.001, **** *p* < 0.0001; ns = no significant difference.

## 3. Results

### 3.1. Characterization and Identification of E. coli and P. mirabilis OMVs

**Figure 1:**
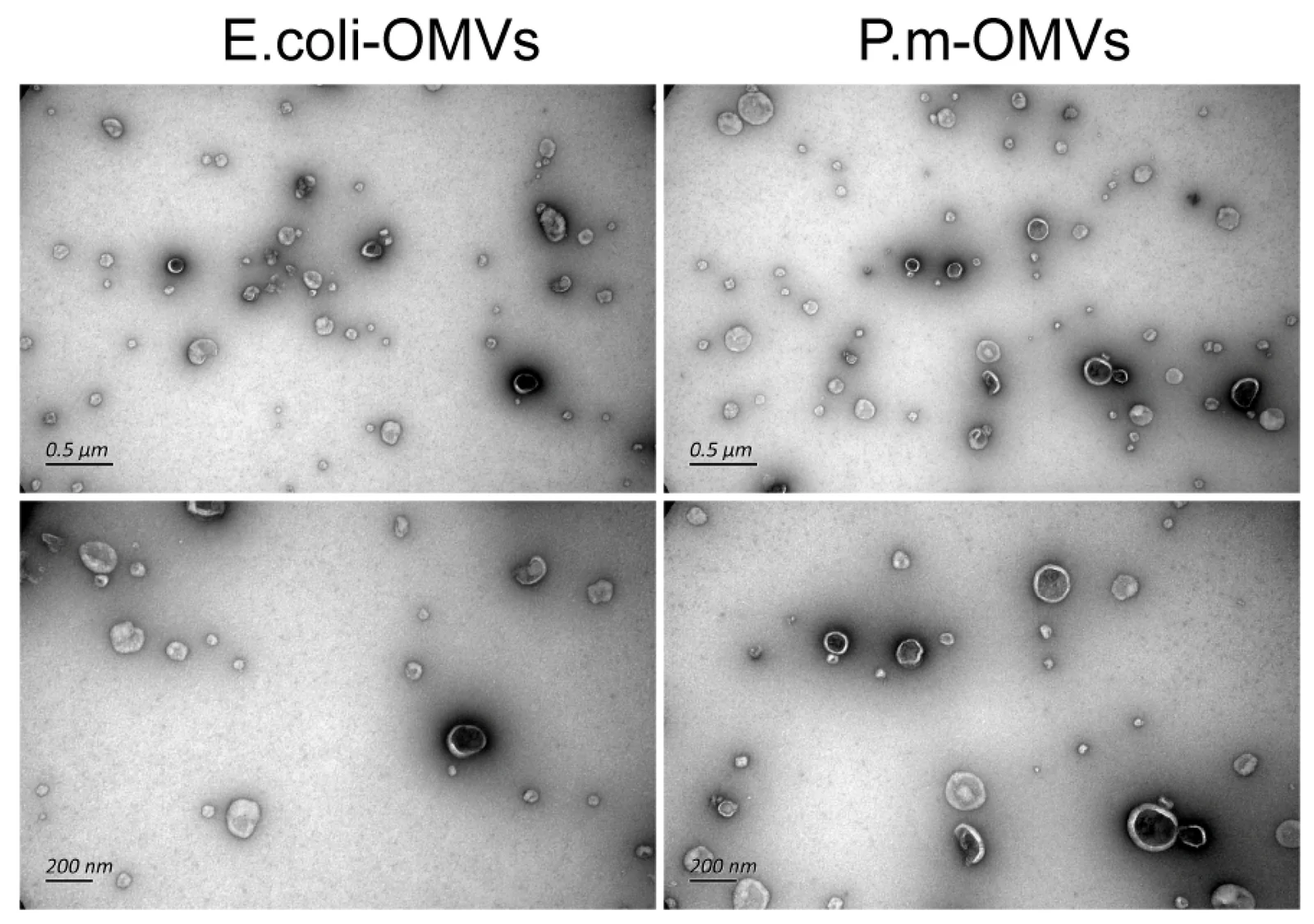
Transmission electron microscopy (TEM) images showing the morphology and structure of *E. coli*-OMVs and *P. m*-OMVs. Scale bar: 200 nm/500 nm (*n* = 3).

**Figure 2:**
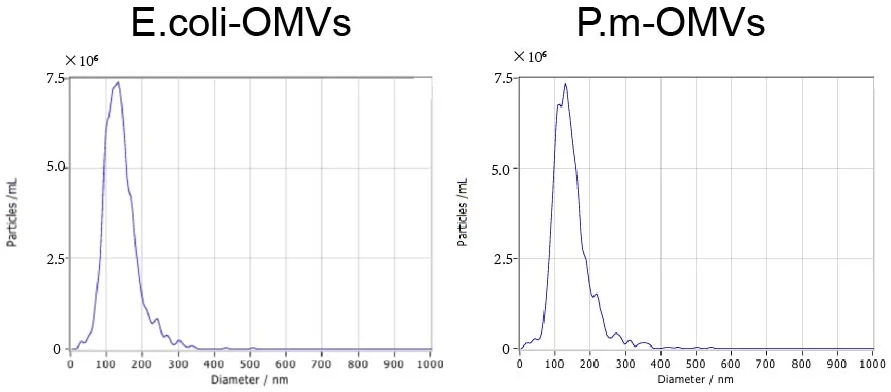
Particle size distribution curves of OMVs isolated from *Proteus mirabilis* and uropathogenic *Escherichia coli* (UPEC). Both X and Y axes are displayed on a linear scale. OMVs from *Proteus mirabilis* had an average diameter of 139.6 nm and a concentration of 3.5 × 10^10^ particles/mL. OMVs secreted by uropathogenic *Escherichia coli* (UPEC) showed an average diameter of 144.0 nm and the same concentration (*n* = 3).

*P. mirabilis*-OMVs and *E. coli*-OMVs, separated by ultracentrifugation, were characterized using transmission electron microscopy (TEM) and nanoparticle tracking analysis. The results showed that both types of OMVs exhibited a typical bilayer membrane vesicle structure (Figure 1). For OMVs isolated from the two bacterial sources, the particle sizes were predominantly between 70 nm and 220 nm, a size range characteristic of OMVs produced by Gram-negative bacteria (Figure 2).

### 3.2. Induction of Cell Damage by OMVs from Both Bacterial Sources, with Similar Trends in Acute Functional Indicators

**Figure 3:**
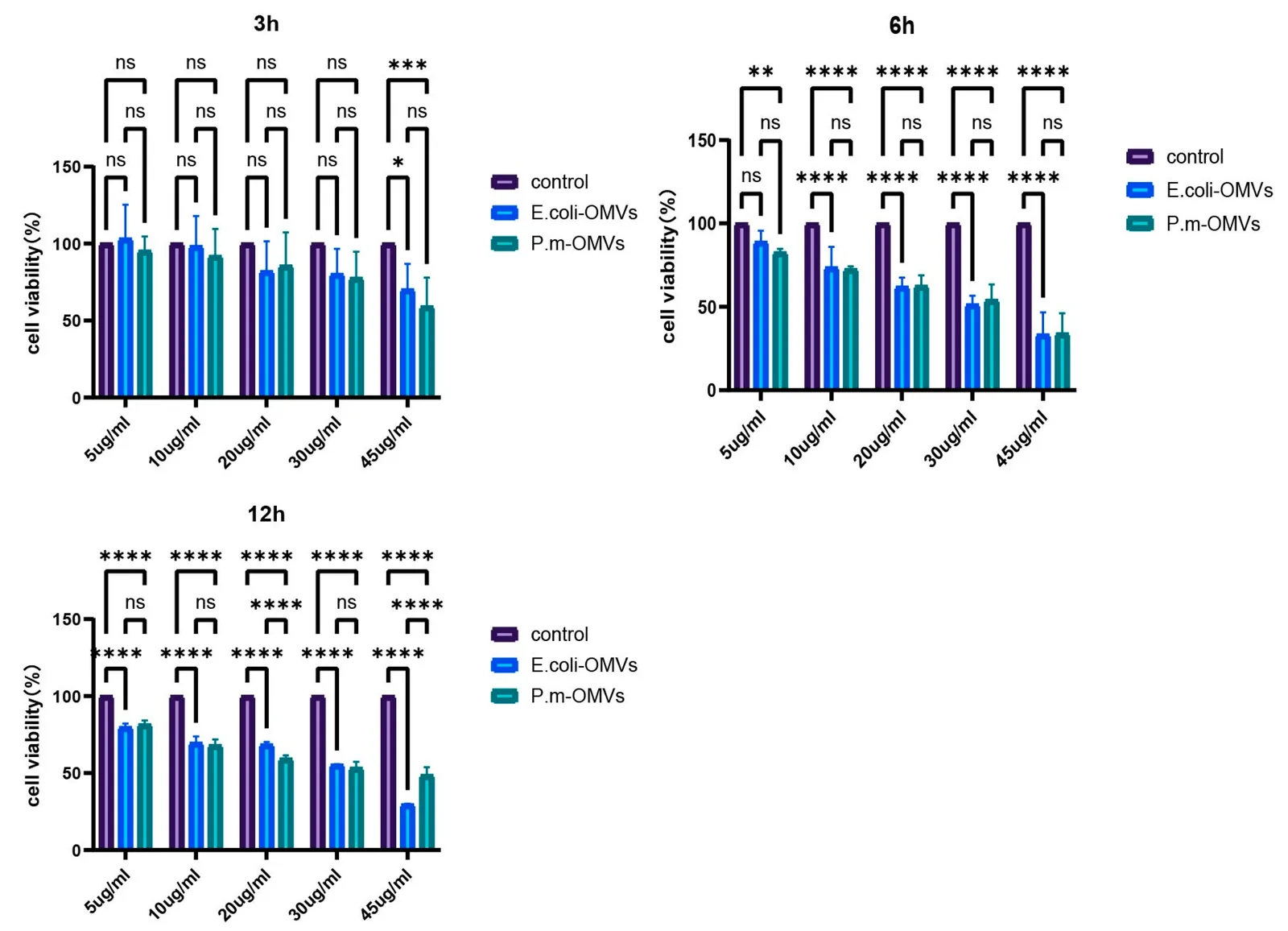
Cell viability curves of HK-2 cells after treatment with *E. coli*-OMVs and *P. mirabilis*-OMVs for 3 h, 6 h, and 12 h. Given the negligible differences in particle size and protein content between *E. coli*-OMVs and *P. mirabilis*-OMVs, protein concentration was used for normalization throughout this study and subsequent experiments. The X-axis represents concentration, and the Y-axis represents cell viability percentage (*n* = 3). Data were analyzed using two-way analysis of variance (ANOVA), followed by Tukey’s post hoc multiple-comparison test. * *p* < 0.05, ** *p* < 0.01, *** *p* < 0.001, **** *p* < 0.0001; ns = no significant difference.

The impact of OMVs on HK-2 cell viability was evaluated using the CCK-8 assay at different time points (3 h, 6 h, and 12 h) and concentrations (5, 10, 20, 30, and 45 µg/mL). The results showed an overall decline in HK-2 cell viability with increasing OMV concentration and exposure time (Figure 3). Notably, after co-culturing HK-2 cells with OMVs for 12 h, a significant reduction in cell viability was observed at 45 µg/mL OMV concentration. Importantly, this condition still permitted subsequent assessments of oxidative stress, membrane permeability, mitochondrial membrane potential, and ultrastructure; therefore, it was selected for the subsequent experiments. Accordingly, HK-2 cells were treated with the two OMV preparations at a concentration of 45 µg/mL (≈8.73 × 10^9^ particles/mL) for 12 h in all subsequent in vitro experiments of this study. Since the particle size and protein content of the two OMVs were similar, protein concentration was used for unified dosing, and particle number was verified for consistency.

### 3.3. Endocytosis of OMVs from Both Bacterial Sources by HK-2 Cells

**Figure 4:**
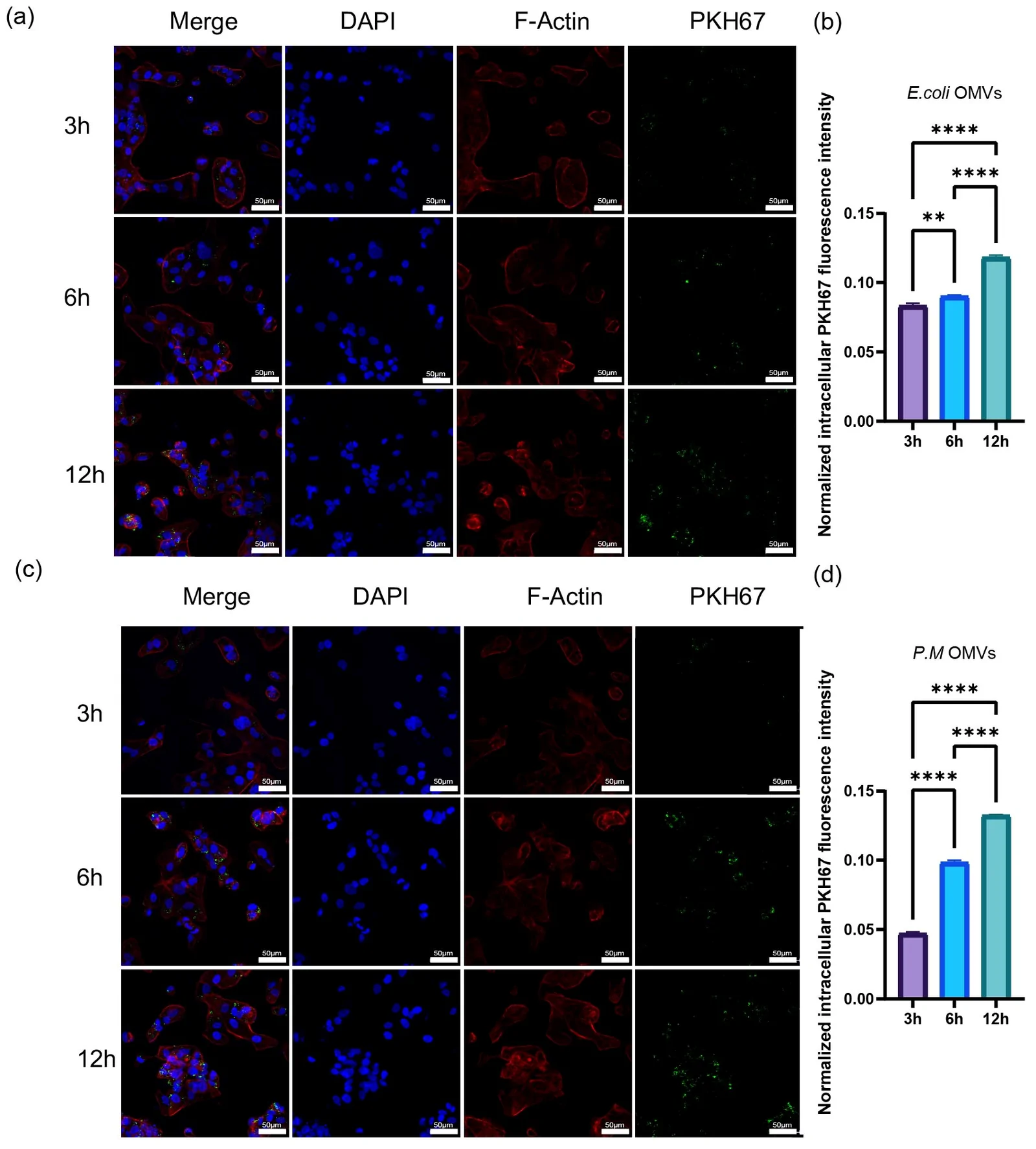
(**a**) Internalization of *E. coli*-OMVs into HK-2 cells observed by laser scanning confocal microscopy at 3 h, 6 h, and 12 h. *E. coli*-OMVs were labeled with PKH67 (green fluorescence), F-actin of HK-2 cells was stained with phalloidin (red fluorescence), and nuclei were counterstained with DAPI (blue fluorescence). (**b**) Quantitative analysis of *E. coli*-OMV internalization. X-axis: incubation time (hours); Y-axis: normalized intracellular PKH67 fluorescence intensity (*n* = 3). (**c**) Internalization of *P. m*-OMVs into HK-2 cells at 3 h, 6 h, and 12 h. (**d**) Quantitative analysis of *P. m*-OMV internalization (*n* = 3).** *p* < 0.01; **** *p* < 0.0001.

Previous studies have revealed that pathogen-derived OMVs can be internalized by host cells to execute their functions [20]. Confocal laser scanning microscopy (CLSM) revealed that PKH-67-labeled *P. mirabilis*-OMVs and *E. coli*-OMVs were both efficiently internalized by HK-2 cells, with clear green fluorescence signals observed intracellularly. This confirms that OMVs from both bacterial sources can enter HK-2 cells via endocytosis. Furthermore, over time, there was a noticeable increase in the number of OMVs within the cells (Figure 4a,c), providing a foundation for subsequent cell injury mediation. Endocytosis assays also confirmed that the amount of internalized OMVs at 12 h was higher than that at 6 h (both *p* < 0.0001, ) (Figure 4b,d).

### 3.4. Induction of Oxidative Stress and Increased Membrane Permeability by OMVs from Both Bacterial Sources in HK-2 Cells

**Figure 5:**
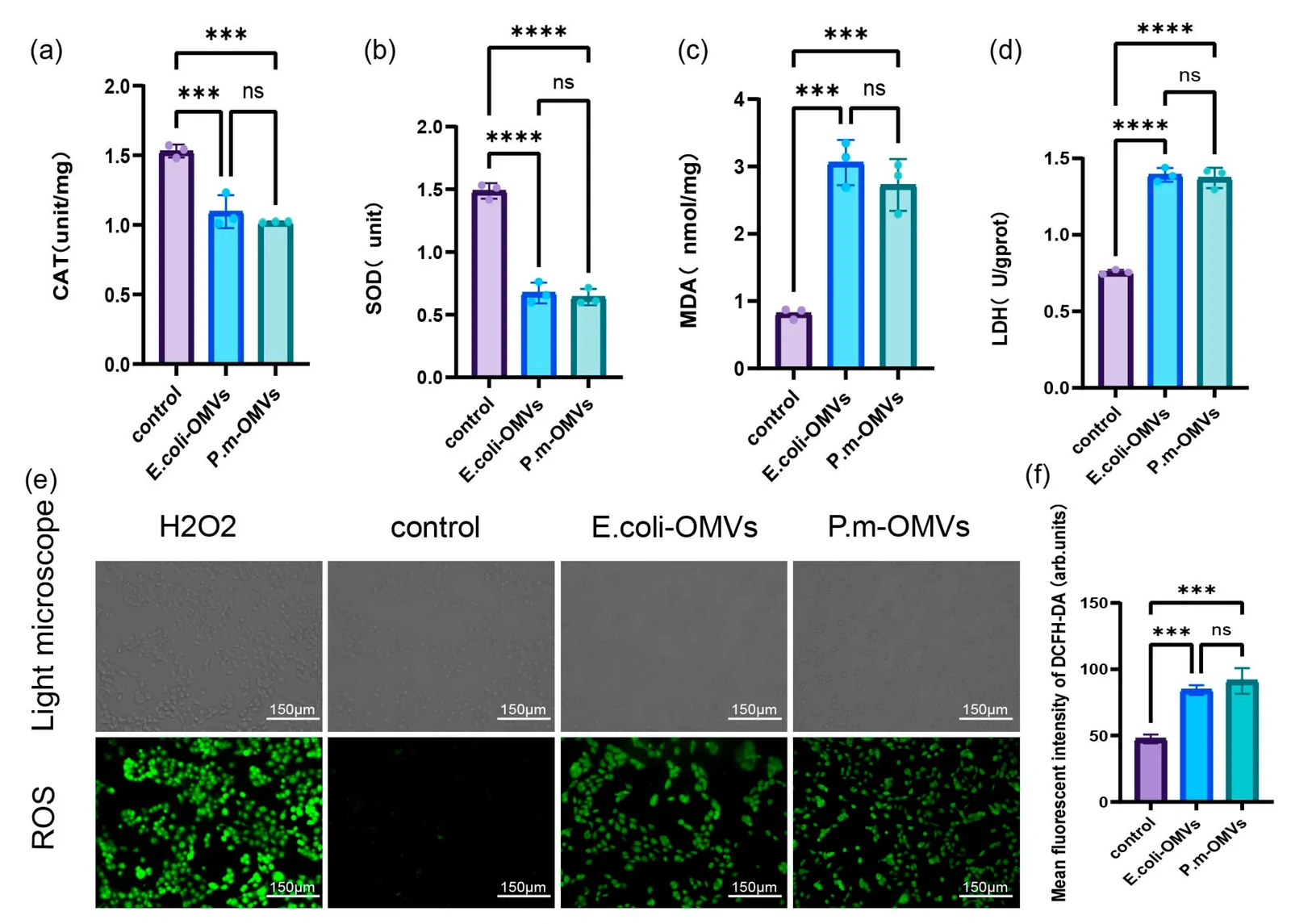
After treatment of HK-2 cells with *E. coli*-OMVs and *P. m*-OMVs for 12 h (control group: normal cells treated with an equal volume of PBS without the addition of bacterial OMVs, to exclude the potential effects of PBS on cells): (**a**) catalase (CAT) levels in HK-2 cells. Compared with the control group, CAT levels were significantly decreased in HK-2 cells treated with *E. coli*-OMVs and *P. mirabilis*-OMVs (*p* = 0.0008, *p* = 0.0004). No significant difference in CAT levels was observed between the two OMV groups (*p* = 0.4799) (*n* = 3). (**b**) Total superoxide dismutase (SOD) activity in HK-2 cells. The total SOD activity was markedly reduced in HK-2 cells after treatment with *E. coli*-OMVs and *P. mirabilis*-OMVs relative to the control group (*p* < 0.0001, *p* < 0.0001). There was no statistically significant difference in SOD activity between the two groups (*p* = 0.8361) (*n* = 3). (**c**) Lipid peroxidation levels in HK-2 cells. Malondialdehyde (MDA) contents were significantly elevated in HK-2 cells exposed to *E. coli*-OMVs and *P. mirabilis*-OMVs compared with the control group (*p* = 0.0002, *p* = 0.0006). No significant difference in MDA levels was found between the two OMV groups (*p* = 0.4136) (*n* = 3). (**d**) Lactate dehydrogenase (LDH) release levels in HK-2 cells. LDH assay results showed that both *E. coli*-OMVs and *P. mirabilis*-OMVs significantly increased LDH release in HK-2 cells (*p* < 0.0001, *p* < 0.0001), and no obvious difference was detected between the two groups (*p* = 0.8642) (*n* = 3). (**e**) Representative images of DCFH-DA staining for intracellular reactive oxygen species (ROS). From left to right: H_2_O_2_ positive control group, control group, *E. coli*-OMVs group, and *P. m*-OMVs group (*n* = 3). (**f**) Quantitative analysis of DCFH-DA fluorescence intensity. The overall reactive oxygen species (ROS) levels in HK-2 cells were significantly increased after treatment with *E. coli*-OMVs and *P. mirabilis*-OMVs when compared with the control group (*p* = 0.0009, *p* = 0.0004). No statistically significant difference in ROS levels was detected between the two OMV groups (*p* = 0.4215) (*n* = 3). *** *p* < 0.001; **** *p* < 0.0001; ns, not significant.

Oxidative stress is a key mechanism underlying cell injury. By assessing the intensity of oxidative stress and membrane permeability, we were able to indirectly gauge the extent of cell damage. Based on the intervention concentration and duration determined in our preliminary experiments, we detected the overall level of reactive oxygen species (ROS), malondialdehyde (MDA), total superoxide dismutase (SOD), catalase (CAT) and lactate dehydrogenase (LDH) in HK-2 cells, so as to clarify the roles of the two types of OMVs in inducing oxidative damage in HK-2 cells. Co-culturing HK-2 cells with OMVs from both bacterial sources for 12 h led to a significant increase in ROS levels (Figure 5e,f), elevated MDA levels (Figure 5c), and a notable reduction in antioxidant enzyme levels, including total SOD (Figure 5b) and CAT (Figure 5a). The increase in LDH levels indicated that both types of OMVs induced oxidative damage in HK-2 cells, with no significant differences between the two bacterial strains (Figure 5d).

### 3.5. Mitochondrial Dysfunction and Structural Damage Induced by OMVs from Both Bacterial Sources in HK-2 Cells, with Specific Ultrastructural Alterations

**Figure 6:**
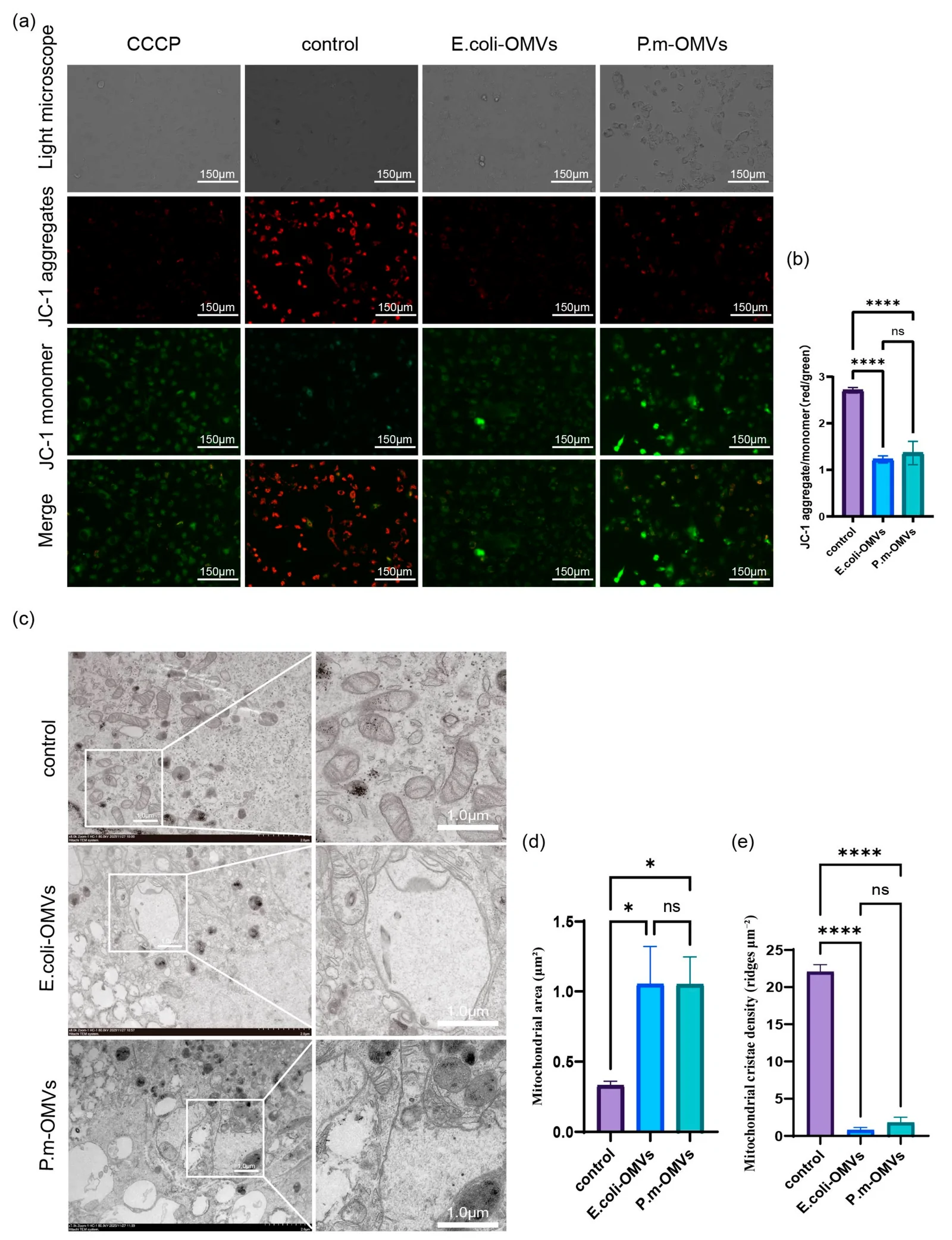
(**a**) Representative images of JC-1 staining showing red fluorescence of JC-1 aggregates and green signals of JC-1 monomers. From left to right: CCCP positive control group, control group, *E. coli*-OMVs group, and *P. m*-OMVs group (*n* = 3). (**b**) Quantitative analysis of the ratio of JC-1 aggregates to JC-1 monomers. Mitochondrial membrane potential in HK-2 cells was markedly decreased following treatment with *E. coli*-OMVs and *P. mirabilis*-OMVs relative to the control group (*p* < 0.0001, *p* < 0.0001). No statistically significant difference was observed between the two OMV groups (*p* = 0.5541) (*n* = 3). (**c**) Representative TEM images showing the morphology of partial organelles in HK-2 cells from the control group, *E. coli*-OMVs group, and *P. m*-OMVs group. Scale bar: 2 µm. (**d**) Quantitative analysis of mitochondrial area. (**e**) Quantitative analysis of mitochondrial cristae number density.* *p* < 0.05; **** *p* < 0.0001; ns, not significant.

Oxidative stress-induced cell damage is closely linked to mitochondrial dysfunction and structural changes. Mitochondrial membrane potential (Δψm) was assessed using JC-1 staining. The results showed that OMVs from both bacterial strains significantly reduced the mitochondrial membrane potential (*p* < 0.0001, *p* < 0.0001), causing mitochondrial dysfunction (Figure 6a,b). However, there were no significant differences in mitochondrial dysfunction between *P. mirabilis*-OMVs and *E. coli*-OMVs after 12 h of HK-2 cell damage (*p* = 0.5541). Interestingly, electron microscopy revealed some distinct structural differences in the mitochondria following treatment with *P. mirabilis*-OMVs and *E. coli*-OMVs. *P. mirabilis*-OMVs caused mitochondrial deformation, a reduction or blurring of mitochondrial cristae. In contrast, *E. coli*-OMVs led to mitochondrial swelling, cristae rupture or disappearance, and vacuolization (Figure 6c). Additional TEM images from independent fields are provided in Supplementary Figure S1. Compared with the control group, mitochondrial area was significantly increased after treatment with *E. coli*-OMVs and *P. mirabilis*-OMVs (*p* = 0.0339, *p* = 0.0345), whereas mitochondrial cristae number density was significantly reduced (*p* < 0.0001, *p* < 0.0001) (Figure 6d,e). No significant differences in mitochondrial area or cristae number density were detected between the two OMV-treated groups (*p* > 0.9999, *p* = 0.5745). Thus, the quantitative measurements confirmed mitochondrial injury in both groups, whereas the differences between the two OMV preparations were primarily reflected in the qualitative patterns of ultrastructural alteration. Although these ultrastructural patterns may provide clues to differences in the cellular injury process, ultrastructural observations alone are insufficient to determine a specific mode of cell death.

## 4. Discussion

Outer membrane vesicles (OMVs) derived from Gram-negative bacteria, as nanoscale natural carriers of virulence factors, can deliver a variety of toxic components (such as lipopolysaccharides [LPS], lipoproteins, nucleic acids, etc.) into host cells upon internalization. This internalization subsequently activates oxidative stress and inflammatory responses, inducing cellular damage [21,22]. The mode of cell injury mediated by bacterial OMVs has been demonstrated to be a crucial component in the pathogenesis of various diseases [6]. This study extends this theory to two common pathogenic bacteria in the urinary system and renal tubular epithelial cells. At the phenotypic level, it confirms that the cytotoxicity mediated by OMVs of these two strains exhibits overall consistency during the acute injury phase.

Mitochondria, as one of the primary sources of reactive oxygen species (ROS) within the cell, play a central role in cellular energy metabolism and the transmission of death signals [23]. The high levels of ROS produced by mitochondrial dysfunction can lead to cellular damage, resulting in loss of cellular function and death [24]. Previous studies have shown that OMVs from Gram-negative bacteria can transfer toxins that inhibit mitochondrial activity, leading to mitochondrial dysfunction, which subsequently causes cellular injury and inflammatory responses [25]. In this study, we further substantiated this viewpoint. Under the same experimental conditions and using the same cell model, we compared the effects of OMVs from two common uropathogenic bacteria on mitochondrial function and ultrastructure in HK-2 cells at the phenotypic level. We observed an intriguing phenomenon: although the mitochondrial membrane potential decreased in all cases during the acute phase of cell injury, there were distinct differences in the mitochondrial ultrastructural damage. Previous studies have primarily focused on the inflammatory pathways induced by OMVs that lead to pyroptosis or apoptosis, with less attention given to the specific organelle damage caused by OMVs from different bacterial species. Our findings suggest that reliance on global functional readouts alone may obscure subtle differences in the pathogenic patterns induced by different pathogens. Organelle ultrastructural changes may therefore provide useful clues for distinguishing the pathogenic patterns of OMVs from different bacterial species.

Both *P. mirabilis*-OMVs and *E. coli*-OMVs exhibited similar cellular injury phenotypes—such as oxidative stress, altered membrane permeability, and mitochondrial dysfunction—after a 12 h exposure. We speculate that this phenomenon may reflect a shared response to multiple virulence factors carried by OMVs, which may activate cellular oxidative stress and inflammatory pathways and ultimately lead to cell damage [26]. Nevertheless, we cannot completely exclude the possibility that nonspecific cellular responses to the internalization of exogenous material contributed to these observations. In the present study, *E. coli*-OMVs cause swelling, cristae rupture, and vacuolization of mitochondria, which are typical manifestations of permeability transition and necrotic damage. These morphological changes are often associated with sustained opening of the mitochondrial permeability transition pore (mPTP), changes in mitochondrial matrix osmotic pressure, and membrane potential collapse [27]. On the other hand, *P. mirabilis*-OMVs induced blurred mitochondrial cristae, accompanied by an apparent increase in lysosome-like structures, a feature that might reflect the cell’s protective response to various stresses, including oxidative stress and mitochondrial damage. On the basis of the mitochondrial morphological differences observed by TEM and the accompanying injury phenotypes, we propose a cautious interpretation of the biological significance of the similar overall acute effects induced by *P. mirabilis*-OMVs and *E. coli*-OMVs. OMVs derived from different uropathogenic Gram-negative bacteria may converge on common cellular stress and injury endpoints. Both preparations share a membranous vesicular architecture and may carry LPS, membrane lipids, proteins, and other bioactive components; these shared features may contribute to convergent phenotypes, including oxidative stress, membrane injury, and mitochondrial dysfunction. Meanwhile, the distinct mitochondrial ultrastructural patterns observed by TEM raise the possibility that the two OMV preparations may engage different upstream cellular processes; however, this interpretation requires direct molecular validation.

This study has several limitations. Although we compared the similarities and differences in the damage phenotypes of HK-2 cells induced by *P. mirabilis*-OMVs and *E. coli*-OMVs, the underlying molecular mechanisms responsible for cellular injury remain to be fully elucidated. Given the complex composition of OMVs, this study only conducted a preliminary investigation, and we did not screen specific virulence factors or conduct proteomic or lipidomic analyses, LPS quantification, or component-fractionation experiments to explain the differential cytotoxic effects of OMVs derived from the two bacterial species. Accordingly, the cargo-based explanation for the shared overall injury phenotypes and the distinct patterns of injury remains hypothetical and requires direct experimental validation. Second, although PBS controlled for the effects of an equal volume of vehicle, the absence of synthetic liposome or mammalian cell-derived exosome controls means that nonspecific effects associated with the internalization of exogenous vesicular material cannot be fully excluded. In addition, as an in vitro cell study, the present work cannot fully recapitulate the complex and dynamic in vivo microenvironment. There are inevitable discrepancies in multiple aspects, including the interactions between bacteria and host/immune cells, as well as the influences of urinary components in vivo. Finally, we only evaluated cellular alterations during the acute injury phase (12 h). The long-term detrimental effects of the two types of OMVs on HK-2 cells still require further investigation. Moreover, molecular markers of apoptosis, pyroptosis, or other forms of cell death, including caspase-3, GSDMD, and IL-1β, were not assessed; therefore, the type of cell death could not be determined. Future studies should integrate OMV proteomics/lipidomics and LPS quantification with transcriptomic or proteomic profiling of HK-2 cells, candidate-component fractionation and functional validation, appropriate non-bacterial vesicle controls, additional urinary tract-relevant cell types, three-dimensional models, organoids, and animal experiments to validate the shared injury phenotypes and distinct mitochondrial morphologies observed here.

## 5. Conclusions

In conclusion, outer membrane vesicles (OMVs) secreted by *Proteus mirabilis* and uropathogenic *Escherichia coli* exerted similar acute damage phenotypes in human renal tubular epithelial HK-2 cells. Nevertheless, the two OMV preparations produced distinct patterns of mitochondrial ultrastructural injury. Whether these differences reflect distinct upstream molecular processes or variations in OMV cargo remains to be determined. Accordingly, when comparing the pathogenic characteristics of different uropathogens, we should not only focus on bacterial cells themselves. OMVs released by these pathogens also serve as indispensable pathogenic factors. This in vitro comparative study further expands the phenotypic characterization of OMV-mediated renal tubular epithelial cell injury. Importantly, this work provides a promising direction for future investigations. Further studies are warranted to elucidate the detailed mechanisms by which virulence factors derived from OMVs of different strains induce divergent damage phenotypes in the same target cells. In addition, it is worthwhile to explore the potential translational value of urinary OMVs as diagnostic biomarkers for urinary tract infections.

## Data Availability

The original contributions presented in this study are included in the article/Supplementary Materials. Further inquiries can be directed to the corresponding author.

## Supplementary Materials

The following supporting information can be downloaded at https://www.mdpi.com/article/10.3390/pathogens15080859/s1: Figure S1: Additional representative transmission electron microscopy images showing mitochondrial ultrastructural changes in HK-2 cells from the control, *E. coli*-OMV, and *P. mirabilis*-OMV groups.

## Abbreviations

The following abbreviations are used in this manuscript:

OMVs	Outer Membrane Vesicles
UPEC	Uropathogenic *Escherichia coli*
*P. m*	*Proteus mirabilis*
LPS	Lipopolysaccharides
ROS	Reactive Oxygen Species
MDA	Malondialdehyde
SOD	Superoxide Dismutase
CAT	Catalase
MMP	Mitochondrial Membrane Potential
LB	Lysogeny Broth
TEM	Transmission Electron Microscopy
CCK-8	Enhanced Cell Counting Kit-8
