# Supplementary material for: Comparative Study of the Cytotoxic Effects of Outer Membrane Vesicles from Proteus mirabilis and Escherichia coli on HK-2 Cells
Source: Pathogens. 2026 Aug 18;15(8):859. doi: 10.3390/pathogens15080859 (PMC13516166; doi:10.3390/pathogens15080859)
Supplement: Supplementary file 1 [file pathogens-15-00859-s001.zip › pathogens-4413989-supplementary.pdf]

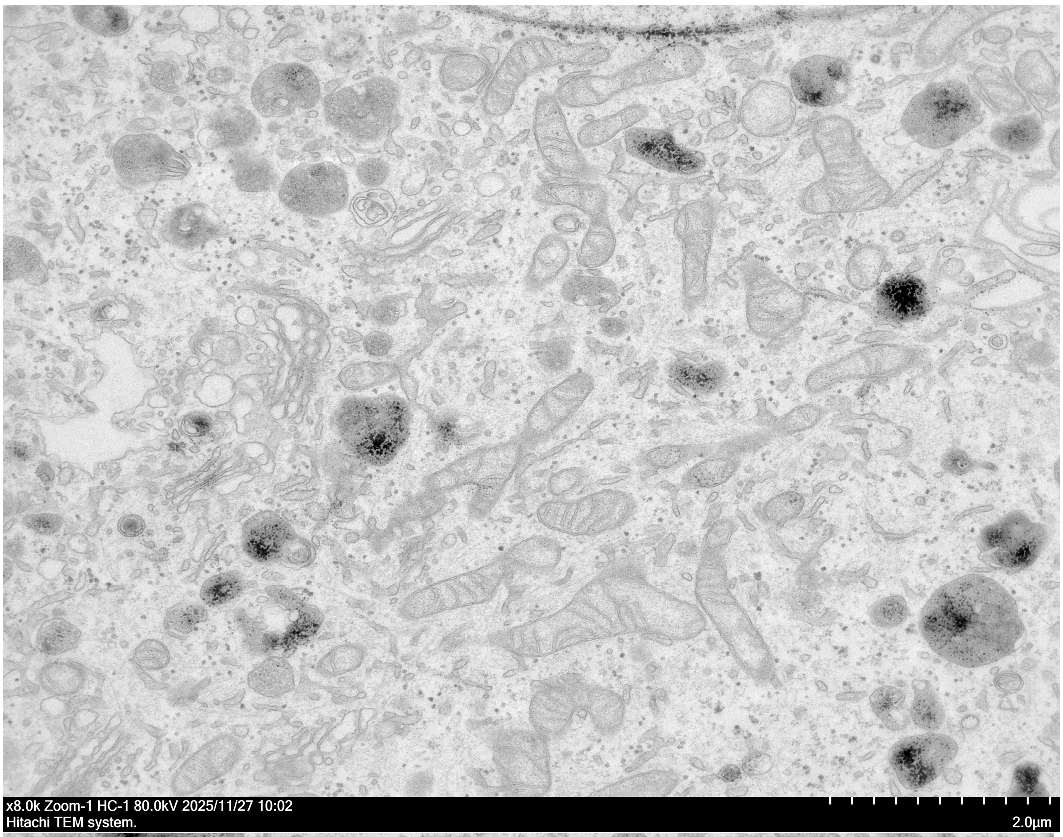
x8.0k Zoom-1 HC-1 80.0kV 2025/11/27 10:02
Hitachi TEM system.
2.0μm

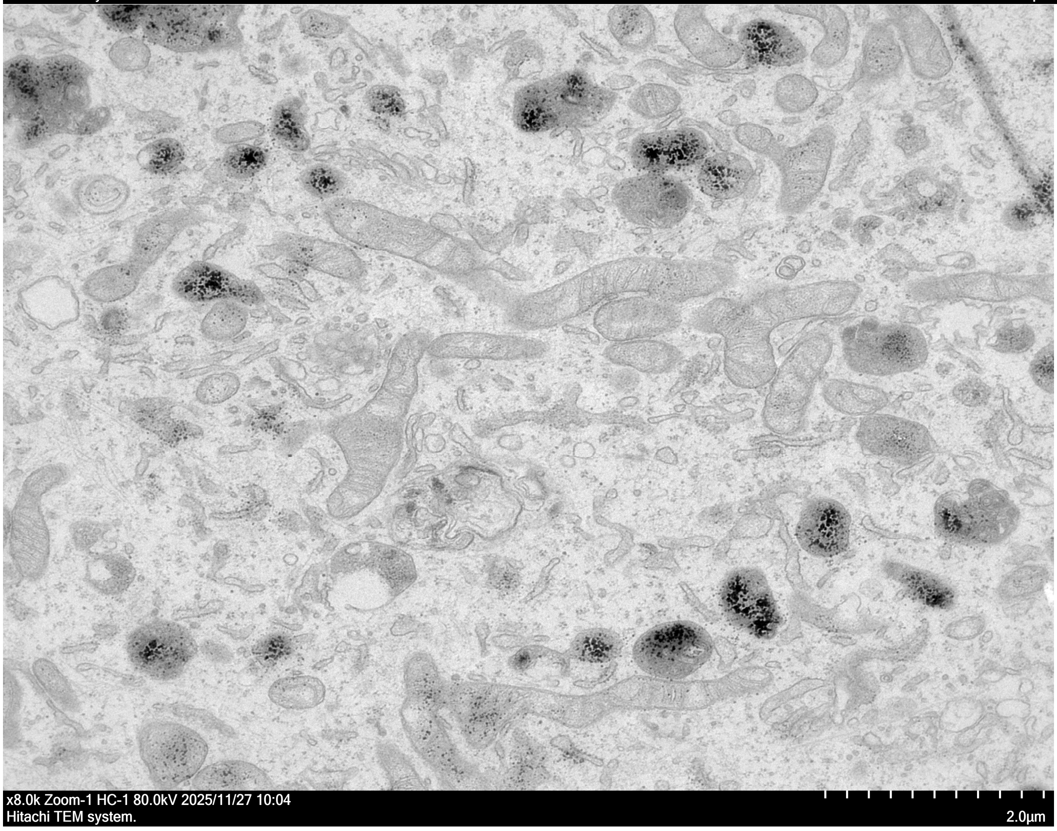
x8.0k Zoom-1 HC-1 80.0kV 2025/11/27 10:04
Hitachi TEM system.
2.0μm

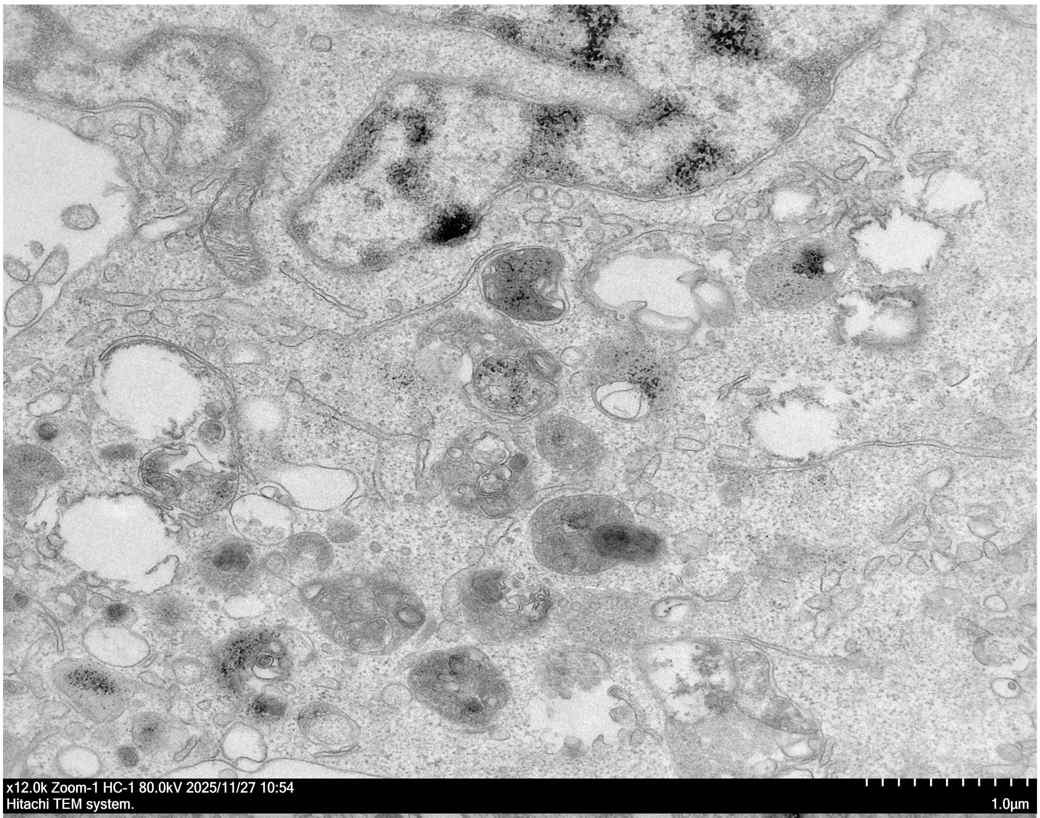
x12.0k Zoom-1 HC-1 80.0kV 2025/11/27 10:54
Hitachi TEM system.
1.0μm

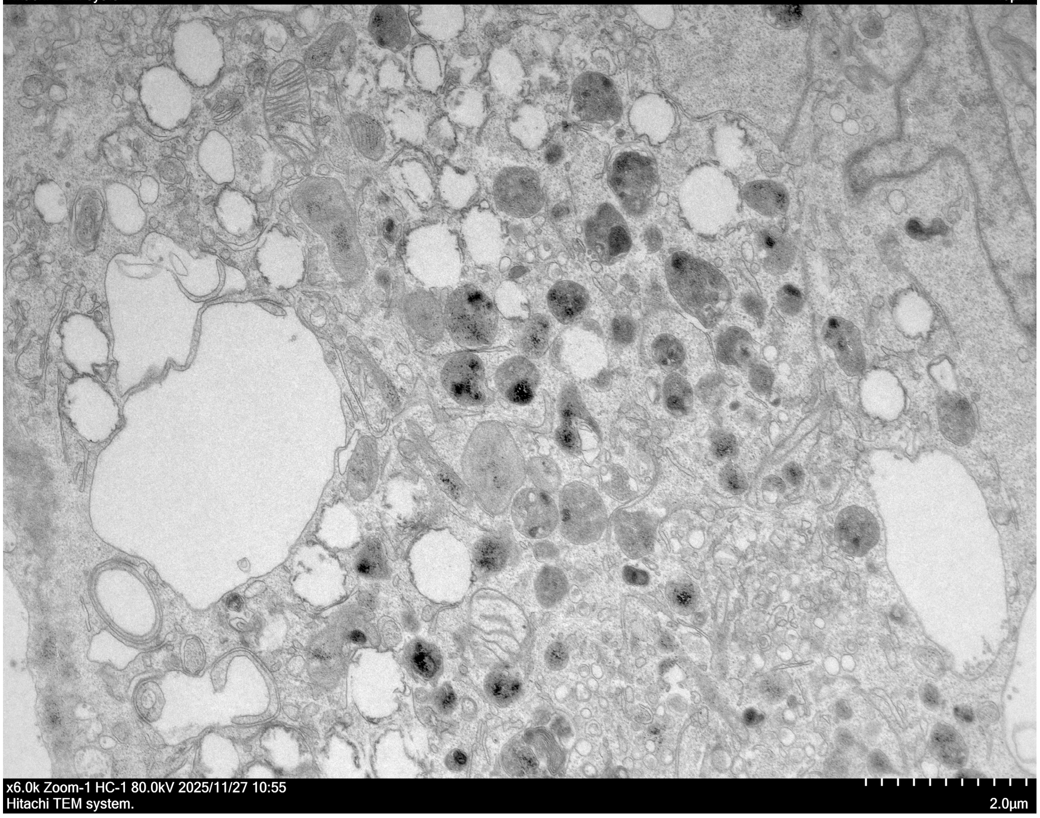
x6.0k Zoom-1 HC-1 80.0kV 2025/11/27 10:55
Hitachi TEM system.
2.0μm

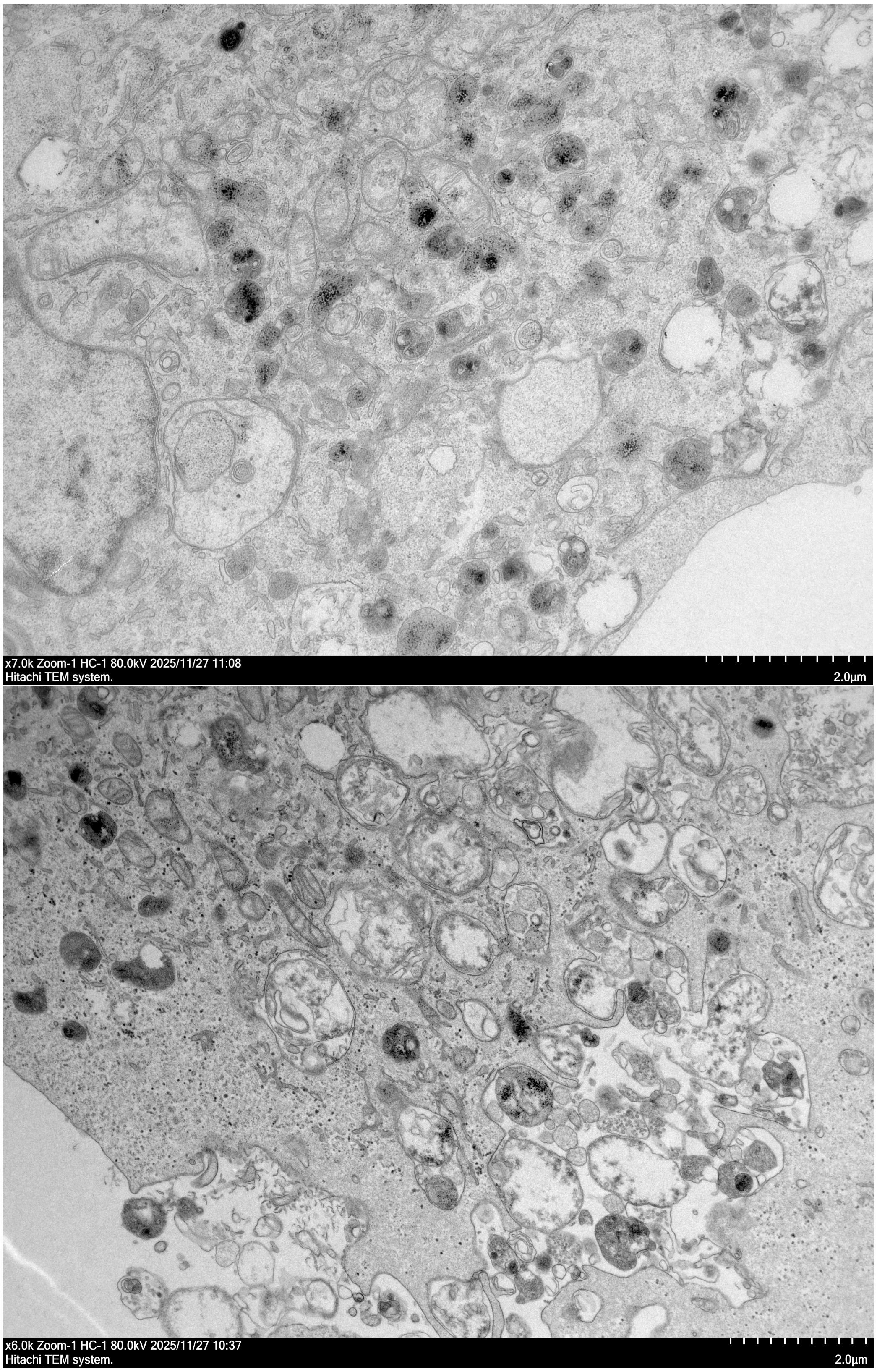

Figure S1. Additional representative transmission electron microscopy images showing mitochondrial ultrastructural changes in HK-2 cells from the control, *E. coli*-OMV, and *P. mirabilis*-OMV groups.
